# Exploring the nematicidal mechanisms and control efficiencies of oxalic acid producing *Aspergillus tubingensis* WF01 against root-knot nematodes

**DOI:** 10.3389/fmicb.2024.1424758

**Published:** 2024-07-08

**Authors:** Zhong-Yan Yang, Yuan-Chen Dai, Yuan-Qi Mo, Jia-Lun Wang, Li Ma, Pei-Ji Zhao, Ying Huang, Rui-Bin Wang, Wei Li, Salim S. Al-Rejaie, Jian-Jin Liu, Yi Cao, Ming-He Mo

**Affiliations:** ^1^State Key Laboratory for Conservation and Utilization of Bio-Resources in Yunnan, Yunnan University, Kunming, China; ^2^Institute of Crop Variety Resources, Guizhou Academy of Agricultural Sciences, Guiyang, China; ^3^Shandong Dianlu Biotechnology Co., Ltd., Feixian, China; ^4^Yunnan Boshiao Biotechnology Co., Ltd., Kunming, China; ^5^Department of Pharmacology and Toxicology, College of Pharmacy, King Saud University, Riyadh, Saudi Arabia; ^6^Pu’er Corporation of Yunnan Tobacco Corporation, Pu’er, China; ^7^Guizhou Academy of Tobacco Agricultural Sciences, Guiyang, China

**Keywords:** *Aspergillus tubingensis*, *Meloidogyne incognita*, oxalic acid, chemotaxis, lignification

## Abstract

**Background and aims:**

Root-knot nematodes (RKN; *Meloidogyne* spp.) are among the highly prevalent and significantly detrimental pathogens that cause severe economic and yield losses in crops. Currently, control of RKN primarily relies on the application of chemical nematicides but it has environmental and public health concerns, which open new doors for alternative methods in the form of biological control.

**Methods:**

In this study, we investigated the nematicidal and attractive activities of an endophytic strain WF01 against *Meloidogyne incognita* in concentration-dependent experiments. The active nematicidal metabolite was extracted in the WF01 crude extract through the Sephadex column, and its structure was identified by nuclear magnetic resonance and mass spectrometry data.

**Results:**

The strain WF01 was identified as *Aspergillus tubingensis* based on morphological and molecular characteristic*s*. The nematicidal and attractive metabolite of *A. tubingensis* WF01 was identified as oxalic acid (OA), which showed solid nematicidal activity against *M. incognita*, having LC_50_ of 27.48 μg ml^–1^. The *Nsy-1* of AWC and *Odr-7* of AWA were the primary neuron genes for *Caenorhabditis elegans* to detect OA. Under greenhouse, WF01 broth and 200 μg ml^–1^ OA could effectively suppress the disease caused by *M. incognita* on tomatoes respectively with control efficiency (CE) of 62.5% and 70.83%, and promote plant growth. In the field, WF01-WP and 8% OA-WP formulations showed moderate CEs of 51.25%–61.47% against RKN in tomato and tobacco. The combined application of WF01 and OA resulted in excellent CEs of 66.83% and 69.34% toward RKN in tomato and tobacco, respectively. Furthermore, the application of WF01 broth or OA significantly suppressed the infection of J2s in tomatoes by upregulating the expression levels of the genes (*PAL*, *C4H*, *HCT*, and *F5H*) related to lignin synthesis, and strengthened root lignification.

**Conclusion:**

Altogether, our results demonstrated that *A. tubingensis* WF01 exhibited multiple weapons to control RKN mediated by producing OA to lure and kill RKN in a concentration-dependent manner and strengthen root lignification. This fungus could serve as an environmental bio-nematicide for managing the diseases caused by RKN.

## 1 Introduction

Plant parasitic nematodes (PPNs) have been estimated to cause global economic losses of >150 billion US dollars annually (Li et al., 2015), and root-knot nematodes (RKN) account for more than half of these losses (Kim et al., 2016). RKN are obligate, sedentary endoparasites of >5,500 plant species (Jaouannet et al., 2012). The RKN infection induces root gall formation, resulting in deficits of essential nutrients and impeding plant growth. Over 100 species of RKN have been reported globally; among them, *Meloidogyne incognita*, *Meloidogyne javanica*, *Meloidogyne arenaria*, and *Meloidogyne hapla* are widely recognized as the most significant species due to their broad host range and heavy economic losses (Elling, 2013). Controlling RKN, compared to other pests, poses a substantial challenge due to their quick multiplication rate and abbreviated reproductive cycle (Adam et al., 2014). During the life cycle of RKN, eggs and second-stage juveniles (J2) are exposed outside the roots, making them the targets of most nematicides. The application of chemical nematicides remains the predominant approach to effectively managing RKN (Antil et al., 2022). Still, their efficacies decrease with prolonged application, increasing environmental and human health risks and causing nematode multidrug resistance (Rajasekharan et al., 2020). In light of the potential health hazards to humans and the environmental challenges posed, it is imperative to employ environmentally friendly and viable approaches to control RKN effectively. Biological control using beneficial microorganisms can serve as a viable, secure, efficient, and feasible approach for RKN control. Various antagonistic organisms, including fungi, bacteria, viruses, mites, turbellarians, collembola, predaceous nematodes, protozoans, etc. have demonstrated effective control against RKN; however, fungi and bacteria remain the predominant biocontrol agents for RKN (Xiang et al., 2018). These microbes can parasitize RKN, reduce J2 invasion, and disrupt egg-hatching by releasing bioactive secondary metabolites. Currently, 12 fungi and 18 bacteria-based bioagents are commercially available for RKN control (Antil et al., 2022).

Nematodes perceive and respond to environmental stimuli through sensory organs, such as amphids and phasmids, which guide them to the source of these stimuli (Tsai et al., 2020). Olfactory chemotaxis toward odors is one of the most robust behaviors of nematodes for their choice of feeding and avoidance of pathogens (Bargmann et al., 1993; Pradel et al., 2007). In contrast, numerous hostile microorganisms possess intricate microcircuits that enable them to counteract olfactory chemotaxis by releasing specialized attractants to deceive and kill nematodes. It has been well established that nematophagous microbes, such as *Pochonia chlamydosporia* (Pacheco et al., 2022), *Esteya vermicola* (Wang et al., 2010), *Streptomyces plicatus* (Wang et al., 2019), and *Pseudomonas putida* (Zhai et al., 2018), can use this olfactory chemotaxis to attract and influence the chemotaxis of PPNs to their hosts through emitting volatile organic compounds (VOCs). The “Trojan horse” mechanism is a remarkable mode of bacterial pathogenesis against nematodes, in which a nematicidal bacterium *Bacillus nematocida* B16 lures nematode *Caenorhabditis elegans* by VOCs (benzaldehyde and 2-heptanone) and kills the nematode by virulent proteases of Bace16 and Bae16 (Niu et al., 2010). However, a similar pathogen-host interaction between nematophagous fungi and RKN is rarely reported.

Nematophagous fungi constitute a significant cohort of natural adversaries to RKN, potentially exerting substantial influence on regulating RKN populations. Over 700 fungal species from the phyla of Ascomycota, Basidiomycota, Chytridiomycota, and Zygomycota, have been demonstrated to display their nematophagous activities by functioning as predators, endoparasites, or toxin-producer (Li et al., 2015). *Aspergillus* is one of the important taxa of filamentous fungi, and its representatives have been found in almost all environments, including plants, soil, marine, and submarine sediments (Zhao et al., 2022). *Aspergillus* spp. produce a plethora of compounds including polyketides, sterols, fatty acids, diphenyl ether, peptides, alkaloids, terpenoids, phenalenones, xanthones, cytochalasins, pyrones, and organic acids (Orfali et al., 2021). These compounds have demonstrated various biological activities, including antibacterial, antifungal, anti-cancer, anti-inflammatory, and cytotoxic effects (Wang P. et al., 2018). In screening of novel nematicidal fungi, we found that the culture filtrates of an endophytic fungus, *Aspergillus* sp. WF01 had markedly nematicidal activity and attractive activity toward nematodes. Additionally, we found that this fungus could increase lignin deposition in the roots of tomatoes. These preliminary findings suggested that the nematicidal mechanism of strain WF01 was possibly different from that of the other *Aspergillus* species reported. Therefore, the aims of this study were (1) to investigate the nematicidal and attractive potential of strain WF01; (2) to characterize the metabolite of strain WF01 involving in nematicidal and attractive activities; (3) to characterize the influence of strain WF01 and its active metabolite on the lignin synthesis and RKN infection in tomato roots; and (4) to evaluate the control efficiencies of strain WF01 formulations on RKN under greenhouse and field conditions.

## 2 Materials and methods

### 2.1 Origin and preparation of nematodes

The RKN used in this study was identified previously as *M. incognita* (Lu et al., 2022). The nematode was maintained on a tomato cultivar (cv. Jiabao), which grew in sand and soil (3:1) at 28°C under a 16/8 h light/dark regime in a glasshouse. Egg masses were picked out from the infected roots with tweezers and placed in a filter paper supported on a square wire gauze in a 6-cm diameter. J2s were collected from hatched eggs after incubation at 28°C for 5–7 days. *C. elegans* N2 (wild-type) and its neuron mutants of AWA (*Odr-7*), ASE (*Che-1*), and AWC (*Nsy-1* and *Odr-1*) were obtained from Caenorhabditis Genetics Center (University of Minnesota, USA). They were cultured and synchronized on an NGM medium with *Escherichia coli* OP50 as a nematode food following the standard methods (van der Most et al., 2023).

### 2.2 Isolation and identification of the strain WF01

Fresh leaves of the medicinal plant *Toddalia asiatica* were collected from Huaxi District, Guiyang City, Guizhou Province, China (106°35′E, 26°23′N). Endophytic fungi were isolated from the leaf following the procedure described by Ma et al. (2013). Among the acquired endophytic fungi, strain WF01 exhibited potent nematicidal activity. The fungus was grown on potato dextrose agar (PDA) plates for 5 days at 28°C for morphological identification. The conidial size and shape were examined under a scanning electron microscope (SEM) (S-3000N, Hitachi, Japan). Fresh mycelia from PDA plates were used for molecular identification using a fungal DNA extraction kit (B518229-0050, Sangon Biotech, China). PCR-mediated amplification of the 5.8S-ITS region with primers ITS1 (5′-TCCGTAGGTGAACCTGCG-3′) and ITS4 (5′TCCTCCGCTTATTGATATGC-3′) was carried out using the procedures described by Yang et al. (2024) and submitted to Beijing Huada Biological Company for sequencing. The ITS sequence of strain WF01 was deposited in GenBank with the accession number ON106833. Existing sequences for reference species were retrieved from GenBank and were aligned using the multiple sequence alignment software CLUSTAL_X v.1.83 (Higgins, 1994). A phylogenetic tree was constructed for obtained sequences using the neighbor-joining method with Kimura’s two-parameter model for calculating genetic distances and 1,000 bootstrap replications using the MEGA 7.0 software.

### 2.3 Identification of the nematicidal metabolite from strain WF01

Fermentation broth (5 L) of strain WF01 was extracted three times with ethyl acetate (3 × 5 L) at room temperature (20–27°C). The organic phase was combined and concentrated to dryness by rotary evaporation at 38°C (NVP-2000, Eyela, Japan) under vacuum to yield crude extracts (12.9 g). The crude extracts were isolated using silica gel G (200–300 mesh) column eluted with a petroleum ether-ethyl acetate (100:1, 50:1, 20:1, 10:1, 5:1, and 0:1) to yield four fractions (WF.1, WF.2, WF.3, and WF.4) (Liu et al., 2022). Fraction WF.2 (26.6 mg) was isolated using a Sephadex LH-20 column eluted with methanol to produce compound A (10.9 mg), which showed the best nematicidal activity. The structure of compound A was identified using data of nuclear magnetic resonance (NMR) and mass spectrometry (MS).

### 2.4 Nematicidal assay of the strain WF01 and oxalic acid

The strain was grown at 28°C on PDA plates for 5 days, and a mycelial disc (5 mm in diameter) was inoculated into a 500 ml Erlenmeyer flask containing 200 ml of potato dextrose broth (PDB). During the 6-day fermentation period at 28°C, 180 rpm, the fermentation was sampled every 24 h for nematicidal assay. Based on the results of the above experiment, the nematicidal metabolite of strain WF01 was identified as oxalic acid (OA). OA (99.99% purity, R093257, Shanghai YiEn Chemical Technology Co., Ltd., China) was dissolved in double distilled water (ddH_2_O) to generate the solution with different concentrations [6.25, 12.5, 25, 50, 100, and 200 μg ml^–1^ for median lethal concentration (LC_50_) determination; 25, 50, 75, 100, 150, and 200 μg ml^–1^ for concentration-dependent analysis].

For the nematicidal assay, 20 μl of nematode suspension (approximately 200 J2s of *M. incognita*) was added into a Cell Culture Dish (Cat. 706001, Nest Biotechnology Co. Ltd., China) containing 1.98 ml of WF01 fermentation or OA solution. A dish containing only PDB and ddH_2_O served as the controls of culture broth and OA, respectively. Nematodes’ mortality rate (MR) was recorded after incubation at 28°C for 24 h. All tests were repeated twice, with three replications of each treatment. Juveniles were judged as dead if their body was straight without movement despite physical stimulation with a fine needle (Kamaruzzaman et al., 2024). The images of J2s in different treatments were taken using a light microscope (SZ-III, Olympus, Japan). MR was corrected using the formula: MR = (MRt-MRc) / (100-MRc) × 100 (Jang et al., 2016). MRt and MRc mean the mortality percentages in treatment and control, respectively. OA concentration and MR data were subjected to probit analysis, and the LC_50_ of OA was calculated.

### 2.5 Chemotaxis assay of the strain WF01 and OA toward nematodes

The WF01 culture broth mentioned above and OA with concentrations of 0.1, 1, and 10 μg ml^–1^ were used in the chemotaxis assay according to the methodology described by Wang et al. (2019). Briefly, the Petri dish (diameter 9 cm) containing 1.5% water-agar medium was divided into three areas: (A) neutral area, located in the center of the plate; (B) test area; and (C) control area. Approximately 100 nematode juveniles (*M. incognita*, the wild-type and mutants of *C. elegans*) in 10 μl ddH_2_O were added to the plate’s center (A). A 10 μl culture broth or OA solution was deposited in position (B), and the same volume of PDB and ddH_2_O was deposited in position (C) as controls, respectively. Plates were placed at 28°C in the dark for 1 h, and the number of nematodes in each area was quantified separately with an inverted optical microscope. Nematodes that remained in the area (A) were not counted. The chemotaxis index (CI) was obtained by subtracting the number of J2s in the test area from the number of J2s in the control area and dividing by the total number of J2 found outside the neutral area (Wang et al., 2019). CI ≥ 0.2 was highly attractive, and 0.1 ≤ CI < 0.2 was slightly attractive. Notably, −0.1 ≤ CI < 0.1, as a random response, −0.2 < CI < −0.1 was considered repellent, CI ≤ −0.2 was highly repellent. The experiment was performed twice, with 10 replicates each.

### 2.6 Evaluation of the efficiencies of strain WF01 and OA against *M. incognita* on tomato under greenhouse conditions

A culture broth of strain WF01 with 6.2 × 10^8^ conidia ml^–1^ was prepared by culturing the strain in PDB at 28°C for 5 days with shaking at 180 rpm. The OA solutions with 100 and 200 μg ml^–1^ concentrations were generated by dissolving the OA into ddH_2_O. The soils used in the pot experiments were collected from the top layer of a tomato-growing field in Eshan, Yunnan, China. Basic physicochemical properties of the soil were determined as (unit: g kg^–1^): total N 4.92, total P 1.31, available P 0.02, total K 15.47, available K 8.31, organic matter 254.63, pH 6.7. After sieving through a sieve (2.5 mm^2^), the soils were mixed thoroughly with commercial peat moss (SAB Germany Gartengold^®^, Syke, Germany) in a ratio of 2:1 (v:v). After autoclaved at 121°C for 2 h, 1 kg of the steam-sterilized mixture in a plastic pot (diameter 15 cm, height 13 cm) was replanted with a tomato seedling (cv. Jiabao, height 15 cm) and drenched with 100 ml of WF01 broth, 100 OA μg ml^–1^, 200 μg ml^–1^, 500-fold dilution of 5% Avermectin^®^ (Veyong Bio-Chemical Co., Ltd., China) or PDB (control). After 3 days of transplantation, 5 ml *M. incognita* suspension (about 2,000 J2s) was inoculated around the roots of each seedling by removing topsoil up to 2–3 cm depth. Each treatment had 30 replicates. Pots were arranged randomly in the greenhouse (25 ± 3°C) and were watered twice a week with 200 ml in a pot. After 60 days of infection, all plants were harvested, and the soils on root surface were washed away with water. The roots were soaked in a beaker filled with water, and the images showing galls on roots were taken. The disease severity of individual plants from each treatment was recorded according on a scale of 0–5, and the disease incidence and control efficiency (CE) were calculated following the description of Huang et al. (2015). Additionally, the plant height and weight were measured.

### 2.7 Influence of strain WF01 and OA on the lignin synthesis and lignin synthesis-related gene expressions in tomato roots

In the above pot experiments, three plants were sampled from the treatment drenched with PDB, WF01 broth, or 100 μg ml^–1^ OA at 2, 4, 7, and 10 days after inoculating the J2s. The root samples were dried in a forced-air oven at 70°C for 24 h and ground into powder. After sieving through a sieve (Aperture = 0.3 mm), 5 mg of the powder was used for lignin content analysis by a lignin assay kit (BC4200, Solarbio, China) according to the instructions provided by the manufacturer. The gene expressions of lignin synthesis-related genes (*PAL*, *C4H*, *HCT*, and *F5H*) were analyzed by the quantitative real-time polymerase chain reaction (qRT-PCR). The total RNA was extracted from 50 mg of fresh tomato root tips in liquid nitrogen using RNAiso Plus reagent according to the manufacturer’s instructions (Takara, Shanghai, China). RNA degradation and contamination were checked on 1% agarose gels, and RNA concentration and purity were monitored using the Thermo Scientific NanoDrop One spectrophotometer (Thermo Fisher Scientific, USA). cDNA was synthesized from 1 μg of total RNA using the Fast King RT Kit (Tiangen, China). The primers used to amplify the lignin genes were described by Veronico et al. (2018). The qPCR was carried out on a Light Cycler 480^®^ Instrument II platform (Roche, Switzerland) with SYBR Green Real-Time PCR Master Mix (Tiangen, China). The qPCR conditions consisted of an initial denaturation at 95°C for 30 s, followed by 40 cycles of denaturation at 95°C for 5 s, annealing at 58°C for 30 s, and extension at 72°C for 30 s. The relative gene expression was calculated by method of 2^–ΔΔCt^ (Livak and Schmittgen, 2001).

### 2.8 Influence of strain WF01 and OA on the invasion of *M. incognita* J2s in tomato roots

In the above gene expression experiments, fresh root tips of tomatoes from treatments of PDB, WF01, and OA also were collected at 2 and 10 days, and used for infection experiments using Pluronic gel (Wang et al., 2022). Briefly, 23 g of Pluronic F-127 powder (Sigma-Aldrich) was dissolved in 77 ml of precooled sterile water at 4°C for 24 h. About 3 ml of Pluronic gel was poured into a Petri dish (3.5 cm diameter). Five root tips with a length of about 1 cm were placed at the center of the Petri dish. About 100 J2s were inoculated at 0.5 cm from each root tip. Every treatment contained three replicates. All dishes were placed in an incubator for 24 h at 28°C. Then, the roots were rinsed with distilled water and stained with acid fuchsin to observe the distribution of nematodes in the roots (Bybd et al., 1983). The number of J2s in the roots was counted under a stereomicroscope (Nikon, Japan).

### 2.9 Evaluation of efficiencies of WF01 and OA against RKN under field conditions

To prepare WF01 formulation, 5 ml conidial suspension (1.5 × 10^6^ conidia ml^–1^) of WF01 was inoculated into a 1 L Erlenmeyer flask containing solid-state medium (wheat bran 75 g, rice bran 75 g, molasses 5 g, peptone 3 g, ddH_2_O 100 ml), followed by incubation at 28°C for 7 days in the dark to yield 7.5 × 10^9^ conidia g^–1^. A formulation of WF01-WP was prepared by mixed solid-state culture of WF01 (100 g) with 720 g of kaoline (K915604, Shanghai Maclin Biochemical Technology Co., Ltd., China), 90 g of sodium dodecyl sulfate (S817788, Shanghai Maclin Biochemical Technology Co., Ltd., China) as a wetting agent, and 90 g of sodium poly sulfonate (S830220, Shanghai Maclin Biochemical Technology Co., Ltd., China) as a dispersal agent (Jang et al., 2016). The mixture was milled in a blender (MYP2011-150, Shanghai Meiyingpu Instrument Manufacturing Co., Ltd., China). The WF01-WP contained a viable conidial count of 6.4 × 10^8^ conidia g^–1^. The OA-WP containing 8% OA was prepared by mixing 80 g of OA, 270 g of white carbon (S915310, Shanghai Maclin Biochemical Technology Co., Ltd., China), 90 g of sodium dodecyl sulfate, 90 g of sodium poly (naphthalene formaldehyde) sulfonate, and 570 g of kaoline (Jang et al., 2016). Meanwhile, a complex formulation (WF01 + OA) was prepared by mixing the WF01-WP with OA-WP at a ratio of 1:1 (W: W).

The field experiments were conducted respectively in tomato and tobacco fields naturally infected by *Meloidogyne* spp., located in Eshan County, Yuxi City, Yunnan Province, China, from April to September 2023. In each field, five treatments were performed: (A) Water (Control), (B) 500-fold dilution of WF01-10%WP, (C) 500-fold dilution of OA-8%WP, (D) 500-fold dilution of WF01 + OA, and (E) 1,000-fold dilution of 5% Avermectin. Each seeding of tomato (cv. Jinfen-2) or tobacco (cv. Hongda) was irrigated with treatment dilutions (100 ml) two times, respectively at 0 and 30 days after transplantation of the seedling. The field experiments were performed using a randomized complete block design. All treatments had three replicates with 200 plants per replicate. The CEs of treatments were calculated as mentioned above at 90 days after transplantation.

### 2.10 Data analysis

SPSS Statistics was used to analyze the data, calculate the standard deviation of each experimental group, and perform the one-way ANOVA. The significant differences were determined based on *p* < 0.05 using the least significant difference (LSD) analysis. The LC_50_ was determined by probit analysis (95% confidence limits) using the SPSS software.

## 3 Results

### 3.1 Strain WF01 was identified as *Aspergillus tubingensis*

**FIGURE 1:**
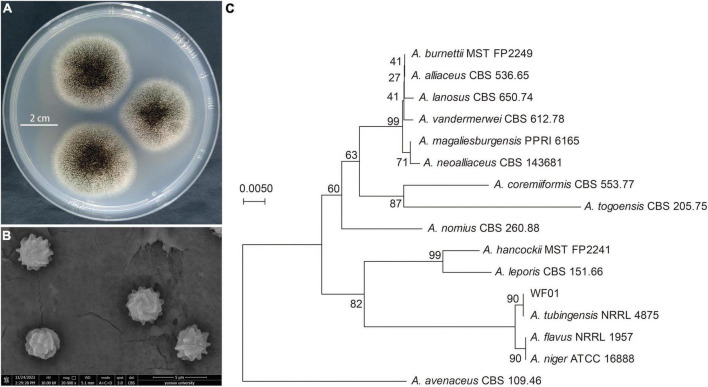
Identification of *A. tubingensis* WF01. **(A)** Colony morphology of strain WF01 on PDA after incubation of 3 days at 28°C. **(B)** Conidial shape of strain WF01 under the scanning electron microscope. **(C)** The ITS-based dendrogram showing the phylogenetic relationship of strain WF01 closely related to *A. tubingensis* NRRL 4875.

Identification of strain WF01 was made based on morphological characteristics such as shape, size, and color of colony and conidia. On PDA medium, WF01 produced black color colonies with white mycelium when sporulating with a diameter of about 4.0–4.5 cm after 3 days of incubation at 28°C (Figure 1A), and no diffusing pigment was observed on the backside. Conidiophores were borne from aerial hyphae, mostly in 730–1,100 × 8.5–11.5 μm in size, with oval vesicles on the tip bearing phialides and conidia around the entire circumference. Conidia were spherical, with a diameter of about 3.3 μm and a very rough surface, as observed in SEM (Figure 1B). Genotypically, the NJ tree supported that the WF01 (Accession: ON106833) belonged to the genus of *Aspergillus* (Figure 1C) and exhibited the highest similarity of 100% to *A. tubingensis* NRRL 4875 (Accession: NR_131293.1) according to 5.8S-ITS rRNA gene sequence analysis. Thus, strain WF01 was identified as *A. tubingensis* based on morphological and molecular analysis results.

### 3.2 Oxalic acid was the nematicidal metabolite of *A. tubingensis* WF01

The nematicidal metabolites were found in the organic phase after extraction of the fermentation broth with ethyl acetate. Four crude extracts were obtained from the organic phase of WF01, and WF.2 was isolated using a Sephadex LH-20 column eluted with methanol to generate compound A, which showed strong nematicidal activity. The structure of A was identified as OA by using NMR and MS data (Furrow and Aurentz, 2010): colorless crystal; C_2_H_2_O_4_; HR-ESI-MS m/z: 88.9864 [M–H]^–^; ^13^C-NMR (150 MHz, CD_3_OD) δ: 161.0 (s).

### 3.3 Strain WF01 and OA showed strong nematicidal activities

**FIGURE 2:**
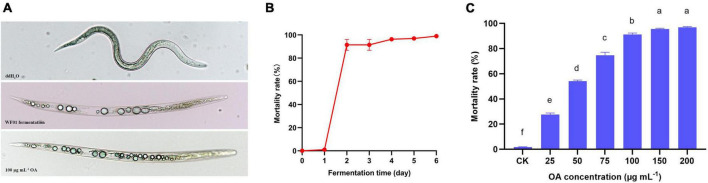
The lethal symptoms and nematicidal activities of *A. tubingensis* WF01 and OA against *M. incognita* J2s. **(A)** The lethal symptoms of J2s caused by WF01 fermentation, 100 μg ml^–1^ OA solution, and ddH_2_O. **(B)** Nematicidal activities of WF01 fermentation at different fermentation times. **(C)** Nematicidal activities of OA solution with different concentrations. Means with the same letter are not significantly different (*p* < 0.05) according to LSD analysis.

As shown in Figure 2A, the formation of multiple vacuoles in the body of dead nematodes was the typical symptom caused by WF01 fermentation and OA solution. During 6 days of fermentation at 28°C and 180 rpm, the nematicidal activity of WF01 fermentation was measured every 24 h by treating the J2s of *M. incognita* for 24 h. The 2-day-old fermentation of WF01 showed a high mortality rate (MR) of 91.48%, and the MRs increased slowly near 100% from 2 to 6 days (Figure 2B). The OA showed its nematicidal activity in a concentration-dependent manner. At the concentrations of 25, 50, 75, 100, 150, and 200 μg ml^–1^, OA solution resulted MRs of 27.62%, 54.09%, 74.76%, 91.33%, 95.61%, and 96.87%, respectively (Figure 2C). The LC_50_ value of OA against *M*. *incognita* was determined as 27.48 μg ml^–1^.

### 3.4 Strain WF01 and OA served as the attractants to *M*. *incognita* and *C. elegans*

**FIGURE 3:**
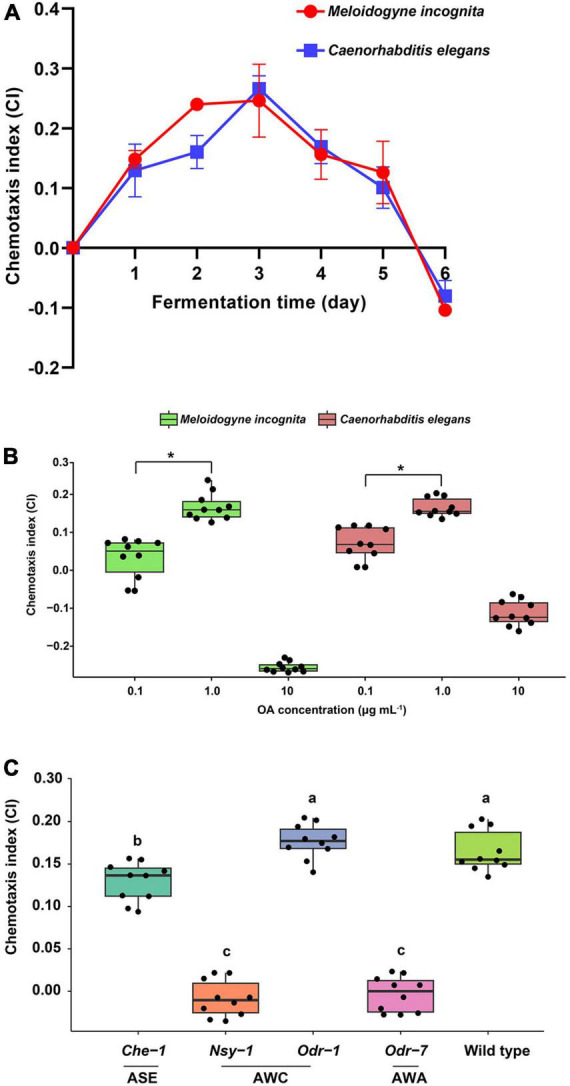
Chemotactic activities of *A. tubingensis* WF01 and OA toward *M. incognita* and *C. elegans*. **(A)** Chemotactic response of nematodes to WF01 broth at different fermentation times. **(B)** Chemotactic response of nematodes to varying concentrations of OA. **(C)** Chemotactic response of *C. elegans* wild-type and its neuron gene mutants to 1 μg ml^–1^ OA. According to LSD analysis, means with the same letter are not significantly different (*p* < 0.05); asterisk denotes statistically significant differences (*p < 0.05).

Chemotaxis of nematodes toward the WF01 fermentation at different fermentation times was analyzed. The WF01 broth showed similar chemotactic intensities toward *M. incognita* and *C. elegans* (Figure 3A). Chemotaxis indexes (CI) increased gradually during the first 3 days of fermentation. The broth displayed highly attractive activities on the third day, where the CI values of both nematodes were >0.2. After that, the chemotactic intensities of the broth gradually decreased to slightly attractive (0.1 ≤ CI < 0.2) from day 4 to day 5, and at day 6, the broth showed repellent activities to the nematodes (CI ≈ −0.1).

The attractive or repellent activities of OA toward *M*. *incognita* and *C. elegans* depended on its concentrations (Figure 3B). At the concentration of 0.1 μg ml^–1^, the CI values of OA to *M*. *incognita* and *C. elegans* were 0.03 and 0.07, respectively, determined as a random response. The OA showed slightly attractive activities (0.1 ≤ CI < 0.2) at the concentration of 1 μg ml^–1^ but showed repellent activities to both nematodes at 10 μg ml^–1^, especially the highly repellent to *M*. *incognita* (CI = −0.26).

### 3.5 Nematode detected OA depending on functions of AWC and AWA olfactory neurons

To determine whether *C. elegans* olfactory neurons were necessary for chemotaxis to OA, chemotaxis to 1 μg ml^–1^ OA was assessed for mutants of the genes encoding AWA, ASE, and AWC. Results suggested that *Nsy-1* of AWC and *Odr-7* of AWA were the primary target genes for the nematode to detect OA because the two mutants showed their CI values near zero when compared to the wild-type, which had a CI of 0.165 (Figure 3C).

### 3.6 Strain WF01 and OA effectively controlled the RKN on tomato and tobacco

**FIGURE 4:**
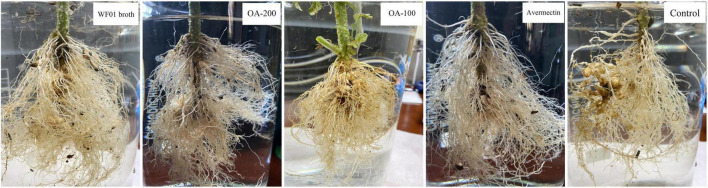
Application of WF01 broth, 200 μg ml^–1^ OA (OA-200), 100 μg ml^–1^ OA (OA-100), and 5% Avermectin (Avermectin) significantly reduced the gall formation caused by *M. incognita* under greenhouse conditions.

**FIGURE 5:**
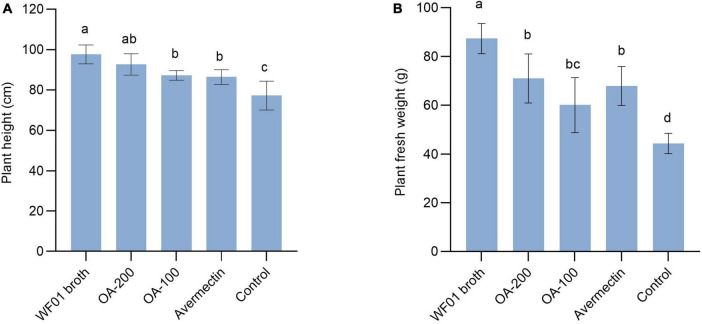
Application of WF01 broth, 200 μg ml^–1^ OA (OA-200), 100 μg ml^–1^ OA (OA-100), and 5% Avermectin (Avermectin) significantly increased the tomato height **(A)** and fresh weight **(B)** in response to *M. incognita* infection. According to LSD analysis, means with the same letter are not significantly different (*p* < 0.05).

Under greenhouse conditions, WF01 culture broth, OA, and Avermectin could markedly reduce the sizes and numbers of galls on tomato roots compared to the control (PDB) (Figure 4). Of the treatments, 200 μg ml^–1^ of OA solution significantly suppressed the infection of *M. incognita* on tomato at a dosage of 100 ml per plant and showed the highest CE same as that of the 5% Avermectin (70.83%) (Table 1). Only application of WF01 broth resulted in a moderate CE (62.5%), which was significantly lower than the treatments of 200 μg ml^–1^ of OA and 5% Avermectin but significantly higher than that of the 100 μg ml^–1^ of OA (41.67%) (*p* < 0.05). Compared to the control, all treatments significantly promoted tomato growth (Figure 5). Inoculation of WF01 culture broth generated the highest values in plant height and fresh weight, while application of 100 and 200 μg ml^–1^ of OA solution showed similar effects on the plant growth as that of 5% Avermectin (Figure 5).

**TABLE 1:** Control efficiencies of the WF01, OA, and their combination against root-knot nematodes under greenhouse and field conditions.

Greenhouse assay on tomato		Field assay on tomato and tobacco		
		Treatment	CE (%)	
**Treatment**	**CE (%)**		**Tomato**	**Tobacco**
WF01 broth	62.50 ± 2.21 b	WF01-WP	57.38 ± 2.14 b	61.47 ± 2.89 b
OA-200	70.83 ± 1.72 a	OA-WP	51.25 ± 1.82 c	57.48 ± 2.61 c
OA-100	41.67 ± 1.13 c	WF01 + OA	66.83 ± 2.86 a	69.34 ± 3.22 a
Avermectin	70.83 ± 3.24 a	Avermectin	68.44 ± 3.17 a	70.21 ± 3.67 a
PDB (control)	–	Water (control)	–	–

The CE values (mean ± SD) in each column followed by the same letter are not significantly different at *p* < 0.05 according to LSD analysis. WF01 broth, WF01 fermentation containing 6.2 × 10^8^ conidia ml^–1^; OA-200, 200 μg ml^–1^ OA solution; OA-100, 100 μg ml^–1^ OA solution; Avermectin, 5% Avermectin^®^; WF01-WP, a wettable formulation containing 10% solid-state culture of WF01; OA-WP, a wettable formulation containing 8% OA; WF01 + OA, a complex formulation containing 50% WF01-WP and 50% OA-WP.

Under field conditions, formulations of WF01-WP, OA-WP, and WF01 + OA respectively resulted in 57.38%, 51.25%, and 66.83% CE on tomato, and 61.47%, 57.48%, and 69.34% CE on tobacco. Remarkably, the WF01 + OA application showed the highest CEs, similar to that of 5% Avermectin on tomato (68.44%) and tobacco (70.21%).

### 3.7 Strain WF01 and OA strengthened root lignification to reduce *M. incognita* infection in tomato

**FIGURE 6:**
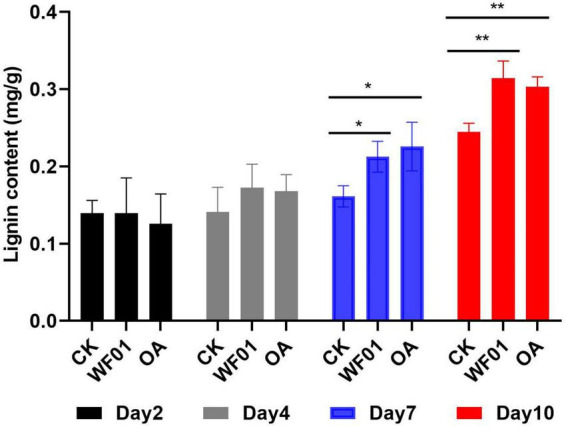
Application of WF01 fermentation (WF01) and 100 μg ml^–1^ OA (OA) significantly increased the lignin contents of tomato roots after treatment for 7 and 10 days in response to *M. incognita* infection. Asterisks denote statistically significant differences (*p < 0.05 and **p < 0.01) according to LSD analysis.

Lignin deposition in the roots of tomatoes from the PDB, WF01 broth treatments, and 100 μg ml^–1^ OA were compared at 2, 4, 7, and 10 days after inoculation of the J2s. As shown in Figure 6, no significant change in the lignin contents was found in the roots among the three treatments at days 2 and 4. However, on days 7 and 10, lignin deposition in roots from the WF01 broth and OA treatments was significantly higher than in the control treated with PDB. At day 7, treatment with OA was more effective in enhancing the lignin content of roots than inoculation of WF01 broth (*p* < 0.05), while WF01 broth was more effective than OA at day 10 (*p* < 0.01).

**FIGURE 7:**
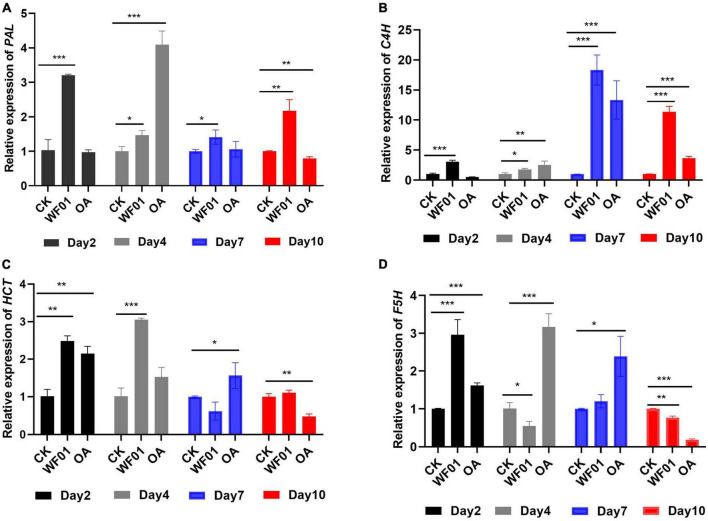
Expression profiles of *PAL* **(A)**, *C4H* **(B)**, *HCT* **(C)**, and *F5H* **(D)** genes in tomato roots in response to *M. incognita* infection and treatment with WF01 fermentation (WF01) or 100 μg ml^–1^ OA (OA). Asterisks denote statistically significant differences (*p < 0.05, **p < 0.01, and ***p < 0.001) according to LSD analysis.

Meanwhile, the expression levels of the genes (*PAL*, *C4H*, *HCT*, and *F5H*) related to lignin synthesis were analyzed at the four sampling time points. qRT-PCR revealed that genes of *PAL* (Figure 7A) and *C4H* (Figure 7B) behaved in a similar expression fashion between the treatments of WF01 and control. The expression of *PAL* and *C4H* were significantly upregulated in WF01-treated plants compared to that in control plants at all investigated time points, in which the highest expression differences of *PAL* and *C4H* occurred at day 2 (3.11-fold) and day 7 (18.32-fold), respectively. Treatment with OA only significantly upregulated *PAL* expression at day 4 (4.07-fold), while upregulated *C4H* expression at all time points except day 2. For gene *HCT* (Figure 7C), the transcript levels of plants treated with WF01 were upregulated at days 2 and 4, then returned to the same levels as the control at days 7 and 10. Treated with OA significantly upregulated *HCT* expression at days 2 and 7, but not days 4 and 10. The transcript accumulation of *F5H* in plants significantly upregulated at the early stage (day 2) when treated with WF01, and upregulated at medial stages (days 4 and 7) when treated with OA (Figure 7D). Overall, the genes’ expressions were upregulated at least at one of the investigated time points when treated with the tomatoes with WF01 or OA.

### 3.8 Strain WF01 and OA could effectively suppress the invasion of *M. incognita* J2s in tomato

**FIGURE 8:**
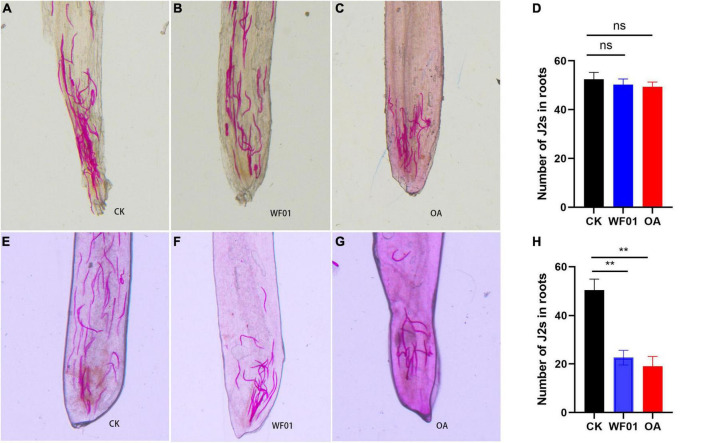
Influence of WF01 fermentation (WF01) and 100 μg ml^–1^ OA (OA) on invasion of *M. incognita* J2s toward tomato roots in Pluronic gel. **(A–C)** Localization of J2s inside the roots respectively treated with PDB (CK), WF01 fermentation (WF01), and 100 μg ml^–1^ OA (OA) at 2 days postinfection. **(D)** No significant difference on the J2 number inside the roots respectively treated with PDB (CK), WF01 fermentation (WF01), and 100 μg ml^–1^ OA (OA) at 2 days postinfection. **(E–G)** Localization of J2s inside the roots respectively treated with PDB (CK), WF01 fermentation (WF01), and 100 μg ml^–1^ OA (OA) at 10 days postinfection. **(H)** The J2 numbers inside the roots respectively treated WF01 fermentation (WF01) and 100 μg ml^–1^ OA (OA) significant lower than that of the PDB (CK) at 10 days postinfection. Asterisks denote statistically significant differences (**p < 0.01) according to LSD analysis.

As shown in Figures 8A–D, the J2 numbers in each root ranged from 49 to 52, and no significant differences in the J2 numbers were found in the tomato plants treated with PDB, WF01 or OA at the sampling time point of day 2. At day 10, the J2s invaded each root of plants treated with WF01 or OA respectively were 23 and 19, which significant lower than that in the control group (50 J2s per root) (*p* < 0.01) (Figures 8E–H). These results suggested that the application of WF01 or OA could effectively suppress the infection of J2s in tomatoes.

## 4 Discussion

As endophytes, *Aspergillus* species exhibited dominance and possessed the capacity to establish colonization within many plant species, including mosses, ferns, liverworts, and hornworts, throughout a wide range of habitats spanning from tropical regions to the tundra (Hagag et al., 2022). These endophytic fungi had demonstrated a diverse array of chemical compounds, such as alkaloids, sterols, diphenyl ether, terpenoids, phenalenones, xanthones, cytochalasins, pyrones, organic acids, as well as butenolides, and these compounds had demonstrated various biological activities, including antibacterial, antifungal, anti-cancer, anti-inflammatory, and cytotoxic effects (Wang P. et al., 2018). Of the *Aspergillus*, some species had demonstrated their nematicidal activities and control potentials against PPNs. In this genus, *Aspergillus niger* was the first reported nematophagous fungus against PPNs through the production of OA (Mankau, 1967). Besides the OA, citric acid also was included in the nematicidal metabolites of *A. niger* against PPNs of *Aphelenchus avenae*, *A. besseyi*, and *M. arenaria* (Zuckerman et al., 1994; Mokbel et al., 2009). *A. niger* exhibited outstanding control potential against RKN mainly through direct nematode eradication via the production of OA (Jang et al., 2016; Lee et al., 2018) and the induction of plant resistance by OA (Yeon et al., 2022). Besides *A. niger*, *Aspergillus welwitschiae*, and *A. tubingensis* were reported as bioagents against RKN. *A. welwitschiae* could decrease the attraction of rice roots to *Meloidogyne graminicola* and suppress the nematode infection by producing the active compound αβ-dehydrocurvularin (Xiang et al., 2020). Previous studies have demonstrated that *A. tubingensis* had a wide range of bioactivities, including the degradation of polyester polyurethane (Khan et al., 2017), the remediation of heavy metal contamination (Shan et al., 2022), the control of plant diseases caused by *Fusarium solani* (Kriaa et al., 2015), and the solubilization of rock phosphates with low solubility (Reddy et al., 2002). Sikandar et al. (2023) first reported the nematicidal activity of *A. tubingensis* against RKN. They found that the fermentation of *A. tubingensis* GX3 broth reduced *Meloidogyne enterolobii* J2 invasion and inhibited nematode development and reproduction in tomato plants. They speculated that *A. tubingensis* GX3 could secrete some form of enzymes or nematicidal materials to destroy eggs and J2s. However, the nematicidal metabolite of this fungus remains unidentified. In the present study, we isolated an endophytic strain WF01 from the leaf of medicinal plant *T. asiatica*, and it was identified as *A. tubingensis* based on its morphological and molecular features. The nematicidal metabolite purified from the fermentation of strain WF01 was identified as OA, representing the initial documentation concerning identifying nematicidal metabolite from *A. tubingensis*.

Strain WF01 demonstrated remarkable nematicidal efficacy against *M. incognita* J2s. The WF01 culture broth resulted in 100% juvenile mortality to *M. incognita* only needed 24 h of exposure time (Figure 2), which was shorter than that of the *A. tubingensis* GX3 to *M. enterolobii* (72 h) (Sikandar et al., 2023). This is possibly due to the differences in strains and target nematodes. The mortality response of WF01 culture broth and OA toward nematodes depended on the fermentation time and OA concentration. Our findings were similar to those of Fan et al. (2020) and Wang et al. (2022), who found a clear correlation between the concentration of the fermentation and active compounds. The efficacy of OA, OA-producing fungus, and their combination in RKN control had been reported by Jang et al. (2016). They discovered that the combined application of *A. niger* F22 and OA to the soil yielded more effective results in managing *M. incognita* on watermelon than using either agent individually. Furthermore, the efficacy achieved through this combination treatment was significantly superior to that of a chemical nematicide known as Sunchungtan (30% fosthiazate). We obtained similar results in our field assay, in which the mixture of *A. tubingensis* WF01 and OA exhibited more success in controlling *M. incognita* in tomato and tobacco plants compared to the individual application of either drug (Table 1). Furthermore, the observed efficacy of strain WF01 and OA combined treatment was found to be comparable to that of the 5% Avermectin. These results suggested that *A. tubingensis* WF01 could serve as an environmentally bio-nematicide for managing RKN diseases.

*Aspergillus* species can generate hydrolytic enzymes, organic acids, and low-molecular-weight natural products that perform a variety of functions, including P solubilization, as well as calcium and iron phosphate solubilization (Cairns et al., 2021). Thus, they have the potential for the development of biofertilizers, which contribute to soil fertility and promote plant growth (Alves et al., 2021). Results from this study indicated that the application of *A. tubingensis* WF01 significantly not only decreased the infection of *M. incognita* but also promoted plant growth (Figure 5). Our findings corroborated the results of Kriaa et al. (2015), Zhao et al. (2018), and Sikandar et al. (2023), who demonstrated that *A. tubingensis* effectively protected plants against phytopathogens and meanwhile increased plant length and fresh mass.

Low-molecular-weight organic acids (LMWOC) are distinguished by the presence of one or more carboxylic groups with a maximum molecular weight of roughly 300 g mol^–1^ (Strobel, 2001). These compounds are generated as metabolic by-products from various organisms and excreted from plant roots. Several LMWOCs as nematicidal metabolites of microorganisms against RKN had been reported. These LMWOCs included the acetic acid from *Paecilomyces lilacinus* and *Trichoderma longibrachiatum* (Djian et al., 1991), kojic acid from *Aspergillus oryzae* (Kim et al., 2016), OA from *A. niger* (Lee et al., 2018), 1,5-dimethyl citrate hydrochloride ester from *Aspergillus japonicus*, 2-furoic acid from *Dactylellina haptotyla* (Lei et al., 2023), trans-aconitic acid from *Bacillus thuringiensi*s (Li and Zhang, 2023), and octanoic acid from *Bacillus altitudinis* (Ye et al., 2022). Many LMWOCs have been reported to show excellent nematicidal activities against RKN. Butyric acid at the 0.1 and 1 mol L^–1^ concentrations could result in 100% mortality of *M. hapla* and *M. incognita* (Browning et al., 2006). The mixture of acetic acid and lactic acid exhibited higher toxicity levels on *M. incognita* J2s than the application of either acid alone (Seo and Kim, 2014). Six volatile fatty acids reduced the rate of egg hatching during 12 days of incubation in the following order: propionic acid > acetic acid > caprylic acid > isobutyric acid > valeric acid > butyric acid (Bansal and Bajaj, 2003). Exposure to 2 mmol L^–1^ OA resulted in 100% juvenile mortality of *M. incognita* and *M. hapla* 1 day after treatment and suppressed egg hatching by 95.6% at 7 days after treatment (Jang et al., 2016). In this study, the LC_50_ of OA against J2s of *M. incognita* was determined as 27.48 μg ml^–1^ at an exposure time of 24 h. The observed value was comparatively lower than the EC_50_ (0.87 mmol L^–1^) reported by Jang et al. (2016), indicating variability in the susceptibility of *M. incognita* J2s to OA across different geographical regions. The formation of multiple vacuoles in the body of dead nematodes is the typical symptom of LMWOCs and their producing microorganisms (Jang et al., 2016; Sikandar et al., 2023; Wang et al., 2023). We also observed the symptoms when *M*. *incognita* J2s were treated with WF01 broth and OA (Figure 2A). The observed phenomenon could possibly be attributed to the high acidity of OA, which effectively deteriorated the cellular and tissue structures of the nematodes. Consequently, this led to disturbances in osmoregulation and subsequent fluid buildup (Seo and Kim, 2014). However, the action mode of LMWOCs toward RKN is still unclear and needs further in-depth studies.

Chemotaxis is a significant behavioral mechanism employed by nematodes to orient their movements in response to specific chemical signals present in their surrounding environment (Zhang et al., 2016). This behavior holds crucial implications for several biological processes, including but not limited to development, predation, food acquisition, metabolism, and signal perception (Hu et al., 2015). Many kinds of organic acids serving as attractants to nematodes have been reported, including hydrochloric acid, sulfuric acid, hypochlorous acid, mesonic acid, acetic acid, formic acid, propionic acid, lactic acid, succinic acid, acetic acid, and citric acid (Wang et al., 2009). One remarkable discovery in the present study was that strain WF01 exhibited the ability to attract juveniles of *M. incognita* and *C. elegans* through the production of OA, with the attraction being dependent on the dosage. During a 6-day fermentation period, the attractive intensities of the WF01 culture broth exhibited an initial increase during the first 3 days, followed by a subsequent reduction leading to a repelling response by day 6 (Figure 3A). This might be due to the OA accumulation in the fermentation broth. The chemotaxis response of OA toward *M. incognita* and *C. elegans* also presented a concentration-dependent manner with 1 μg ml^–1^ for attraction and 10 μg ml^–1^ for repellent (Figure 3B). Compounds attracting or repelling nematodes in a concentration-dependent manner have been reported previously. At 0.5–2.0 mM concentrations, lauric acid attracted *M. incognita* with an increase in the CI values from 0.17 to 0.22, while 4.0 mM lauric acid significantly repelled J2s (Dong et al., 2014). Salicylic acid strongly attracted *M. incognita* at 20–200 μg ml^–1^ concentrations but showed nematicidal activity at high concentrations with LC_50_ of 46 μg ml^–1^ (Wuyts et al., 2006). OA attracted *M. incognita* J2s at 0.1 mg ml^–1^ (Wang et al., 2021) but exhibited substantial nematicidal properties at 2 mmol L^–1^ (Jang et al., 2016).

It has been reported that *C. elegans* exhibits chemotaxis toward various chemicals facilitated by its olfactory neurons. In most cases, *C. elegans* detect attractive compounds emitted by food mainly using the olfactory neurons AWA and AWC (Bargmann, 1993; Zhang et al., 1997), while AWB and ASH neurons are responsible for sensing repulsive volatile chemicals produced by the pathogens (Bargmann, 1998). To further investigate the mechanism by which the strain WF01 attracts nematodes, we employed olfactory neuron mutants of the model organism *C. elegans* as our experimental subjects. This was selected because WF01 culture broth and OA exhibited comparable attractive properties toward *M. incognita* and *C. elegans*. The results of the chemotaxis assay displayed that Odr-7 of AWA and Nsy-1 of AWC were the primary neuron genes for *C. elegans* to detect low concentrations of OA (Figure 3C). It was reported that *C. elegans* could sense the 2-heptanone, a metabolite from the nematicidal bacterium *B. nematocida* B16, depending on the AWC neurons’ function (Zhang et al., 2016). Interestingly, some soil bacteria could exploit nematode chemotaxis to enhance their pathogenicity. This was achieved by releasing certain compounds that served as attractants, effectively enticing nematodes toward them. Subsequently, bacteria could eliminate the nematodes through direct infection or by releasing nematicidal volatiles. For example, *B. nematocida* B16 killed *C. elegans* in the “Trojan horse” mode (Niu et al., 2010). *Paenibacillus polymyxa* KM2501-1 controlled *M. incognita* by the “honey-trap” mode, in which the bacterium released furfural acetone and 2-decanol functioned as “honey-traps” to attract *M. incognita*, and then killed it by other VOCs (Cheng et al., 2017). In this study, *A. tubingensis* WF01 controlled *M. incognita* by a similar mode of action with a low concentration of OA as a “honey-trap” and with a high concentration of OA as a nematicidal weapon. To our knowledge, this is the first report on the “honey-trap” mode involved in the pathogenesis of *Aspergillus* species against nematodes.

Cell wall strengthening through lignin deposition is one of the first barriers used to counteract the pathogen attack (Nicholson and Hammerschmidt, 1992). Lignin drastically modified cell wall structure and functions providing a physical barrier against nematode attack (Holbein et al., 2016). In the present study, we found that treatment with strain WF01 fermentation and OA significantly suppressed the infection of *M. incognita* J2s in tomatoes by upregulating the expression levels of the genes related to lignin synthesis (*PAL*, *C4H*, *HCT*, and *F5H*), and then strengthened root lignification (Figures 6–8). Our findings corroborated Huang et al. (2016), Veronico et al. (2018), and Wang Q. et al. (2018), who reported that lignification of roots played a critical role in increasing plant resistance and then reducing RKN infection.

## 5 Conclusion

In summary, this study presented an excellent nematophagous fungus, *A. tubingensis* WF01, by producing the active metabolite of OA. Fermentation of *A. tubingensis* WF01 and OA exhibited nematicidal and attractive activities to *M. incognita* in a concentration-dependent manner. The *Nsy-1* of AWC and *Odr-7* of AWA were the main neuron genes for nematodes to detect OA. Application of *A. tubingensis* WF01 and OA significantly suppressed the infection of J2s in tomato by upregulating the expression levels of the genes (*PAL*, *C4H*, *HCT*, and *F5H*) related to lignin synthesis, and then strengthened root lignification. Application of *A. tubingensis* WF01 alone or in combination with OA could effectively control RKN in tomato and tobacco and promote plant growth. Remarkably, the combination of strain WF01 and OA achieved excellent efficacies similar to that of 5% Avermectin, a commercial nematicide widely used worldwide. Conclusively, the OA-producing *A. tubingensis* WF01 exhibited multiple weapons to control RKNs, which included luring and killing the nematodes by OA in the manner of concentration-dependent, and strengthening root lignification. This fungus could serve as an environmental bio-nematicide for managing the diseases caused by RKN.

## Data availability statement

The raw data supporting the conclusions of this article will be made available by the authors, without undue reservation.

## Author contributions

Z-YY: Data curation, Investigation, Methodology, Writing-original draft. Y-CD: Data curation, Investigation, Writing-original draft. Y-QM: Investigation, Methodology, Writing-original draft. J-LW: Investigation, Methodology, Writing-original draft. LM: Investigation, Methodology, Writing-original draft. P-JZ: Methodology, Writing-original draft. YH: Investigation, Methodology, Writing-original draft. R-BW: Investigation, Methodology, Writing-original draft. WL: Investigation, Methodology, Writing-original draft. SA-R: Writing – review & editing. J-JL: Funding acquisition, Writing-original draft, Writing – review & editing. YC: Funding acquisition, Writing – review & editing. M-HM: Conceptualization, Funding acquisition, Supervision, Writing – review & editing, Writing – original draft.
